# Vital Signs: Births to Teens Aged 15–17 Years — United States, 1991–2012

**Published:** 2014-04-11

**Authors:** Shanna Cox, Karen Pazol, Lee Warner, Lisa Romero, Alison Spitz, Lorrie Gavin, Wanda Barfield

**Affiliations:** 1 Division of Reproductive Health, National Center for Chronic Disease Prevention and Health Promotion, CDC

## Abstract

**Background:**

Teens who give birth at age 15–17 years are at increased risk for adverse medical and social outcomes of teen pregnancy.

**Methods:**

To examine trends in the rate and proportion of births to teens aged 15–19 years that were to teens aged 15–17 years, CDC analyzed 1991–2012 National Vital Statistics System data. National Survey of Family Growth (NSFG) data from 2006–2010 were used to examine sexual experience, contraceptive use, and receipt of prevention opportunities among female teens aged 15–17 years.

**Results:**

During 1991–2012, the rate of births per 1,000 teens declined from 17.9 to 5.4 for teens aged 15 years, 36.9 to 12.9 for those aged 16 years, and 60.6 to 23.7 for those aged 17 years. In 2012, the birth rate per 1,000 teens aged 15–17 years was higher for Hispanics (25.5), non-Hispanic blacks (21.9), and American Indians/Alaska Natives (17.0) compared with non-Hispanic whites (8.4) and Asians/Pacific Islanders (4.1). The rate also varied by state, ranging from 6.2 per 1,000 teens aged 15–17 years in New Hampshire to 29.0 in the District of Columbia. In 2012, there were 86,423 births to teens aged 15–17 years, accounting for 28% of all births to teens aged 15–19 years. This percentage declined from 36% in 1991 to 28% in 2012 (p<0.001). NSFG data for 2006–2010 indicate that although 91% of female teens aged 15–17 years received formal sex education on birth control or how to say no to sex, 24% had not spoken with parents about either topic; among sexually experienced female teens, 83% reported no formal sex education before first sex. Among currently sexually active female teens (those who had sex within 3 months of the survey) aged 15–17 years, 58% used clinical birth control services in the past 12 months, and 92% used contraception at last sex; however, only 1% used the most effective reversible contraceptive methods.

**Conclusions:**

Births to teens aged 15–17 years have declined but still account for approximately one quarter of births to teens aged 15–19 years.

**Implications for public health practice:**

These data highlight opportunities to increase younger teens exposure to interventions that delay initiation of sex and provide contraceptive services for those who are sexually active; these strategies include support for evidence-based programs that reach youths before they initiate sex, resources for parents in talking to teens about sex and contraception, and access to reproductive health-care services.

## Introduction

The U.S. teen birth rate has continued to decline, from 84.1 births per 1,000 teens aged 15–19 years in 1991 to an all-time low of 29.4 in 2012 (1). Despite this trend, approximately 305,000 infants were born to teens aged 15–19 years in 2012 (1), and the U.S. teen birth rate remains higher than in other developed countries (2). Of particular concern are births to younger teens (those aged 15–17 years), who are not yet legally recognized as adults and are at greatest risk for poor medical, social, and economic outcomes (3). Teens in this age group typically have not completed high school and are subject to state-based limitations on driving and obtaining employment (4,5). Previous research from the National Longitudinal Survey of Youth indicates that teens who gave birth before age 18 years were markedly less likely to earn a high school diploma or general equivalency degree compared with older teens who gave birth (6).

Given the demonstrated impact of early teen childbearing, CDC analyzed data from the natality files of the National Vital Statistics System to better understand patterns of childbearing among this age group, and from the National Survey of Family Growth (NSFG) to describe sexual experience, contraceptive use, and receipt of prevention opportunities among female teens aged 15–17 years.

## Methods

U.S. natality files are compiled annually by CDC’s National Center for Health Statistics and include demographic information, such as maternal age, race, and Hispanic ethnicity for all births in all 50 states and the District of Columbia. This report includes national data from 1991–2012 and state-specific data for 2012 (7).

NSFG is an in-person, household survey that uses a stratified, multistage probability sample of females and males aged 15–44 years to create nationally representative estimates of sexual behaviors, attitudes, and contraceptive use (8). The 2006–2010 sample was used for this analysis; data were restricted to never-married female teens aged 15–17 years. Respondents who reported ever having vaginal intercourse were considered sexually experienced. Respondents who had sex in the 3 months before the time of survey were considered currently sexually active.

Contraceptive use was categorized by level of effectiveness for pregnancy prevention based on the percentage of females who experience pregnancy during the first year of typical use (9). The most effective reversible methods (failure rate <1%) included hormonal implants and intrauterine devices (IUDs), classified as long-acting reversible contraceptives (LARCs); moderately effective methods (failure rate 6%–12%) included pill, patch, ring, or injectable contraception; and less effective methods (failure rate ≥18%) included condom, cervical cap, sponge, rhythm method, withdrawal, and responses marked as “other.” No teens in this age group reported diaphragm use or surgical sterilization. Respondents who used more than one method were categorized according to the most effective method reported. Contraceptive use at last sex and use of clinical birth control services were evaluated among those at risk for pregnancy (i.e., those who were currently sexually active and excluding those who were sterile or were pregnant at the time of last sex). Receipt of clinical birth control services in the past 12 months included receiving counseling about birth control, a checkup or medical test related to birth control, or a method or prescription for birth control.

Self-reported formal sex education and parent communication about birth control and how to say no to sex were evaluated. Data on receipt of formal sex education before first sex also were analyzed.

Differences across racial/ethnic and age subgroups were assessed using chi-square tests without adjustment for multiple comparisons. Differences were considered statistically significant at p<0.05. All analyses were conducted using statistical software to account for the complex sample design of the NSFG.

## Results

**FIGURE 1:**
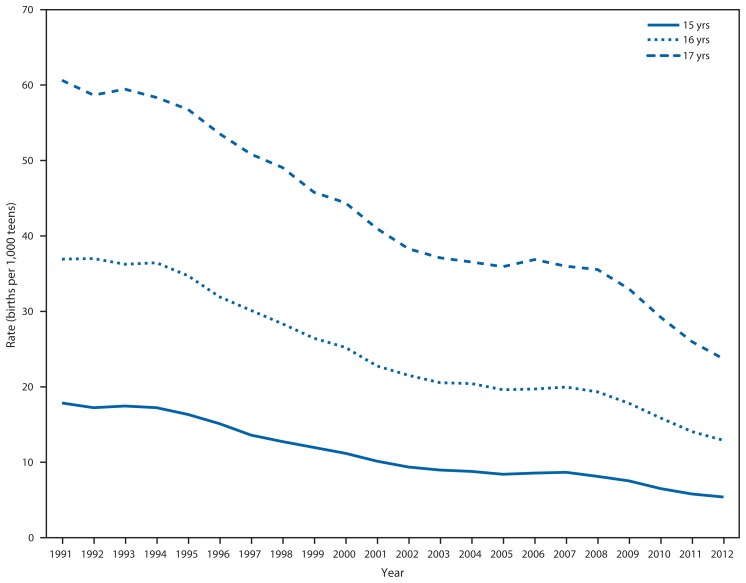
Birth rates for teens aged 15–17 years, by age — United States, 1991–2012 **Source:** CDC’s National Center for Health Statistics. National Vital Statistics System data.

The rate of births per 1,000 teens aged 15–17 years declined 63%, from 38.6 in 1991 to 14.1 in 2012 (1). From 1991 to 2012, the rate of births per 1,000 teens declined from 17.9 to 5.4 for those aged 15 years, 36.9 to 12.9 for those aged 16 years, and 60.6 to 23.7 for those aged 17 years (Figure 1). The percentage decline was higher among teens aged 15 years (70%) compared with those aged 16 years (65%) and 17 years (61%).

**FIGURE 2:**
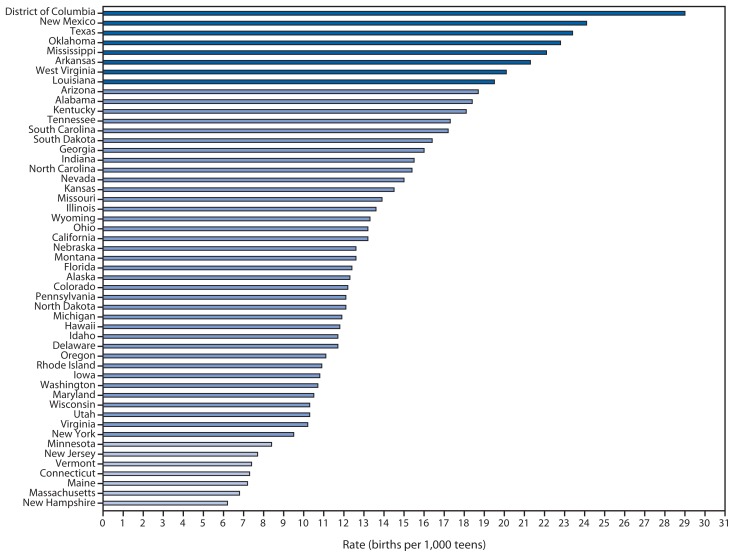
Birth rates for teens aged 15–17 years, by reporting area — United States, 2012^*^ **Source:** CDC’s National Center for Health Statistics. National Vital Statistics System data. ^*^ The rates in reporting areas shaded medium blue are within 1 standard deviation (SD) of the mean. Rates in reporting areas shaded dark blue are >1 SD above the mean. Rates in reporting areas shaded light blue are >1 SD below the mean.

In 2012, the birth rate per 1,000 teens aged 15–17 years varied by race/ethnicity, with the highest rates among Hispanics (25.5), followed by non-Hispanic blacks (21.9), American Indians/Alaska Natives (17.0), non-Hispanic whites (8.4), and Asians/Pacific Islanders (4.1) (1). The rate of births to teens aged 15–17 years also varied widely by state (Figure 2), with the highest rate in the District of Columbia (29.0 per 1,000 teens aged 15–17) and the lowest rate in New Hampshire (6.2) (1).

**FIGURE 3:**
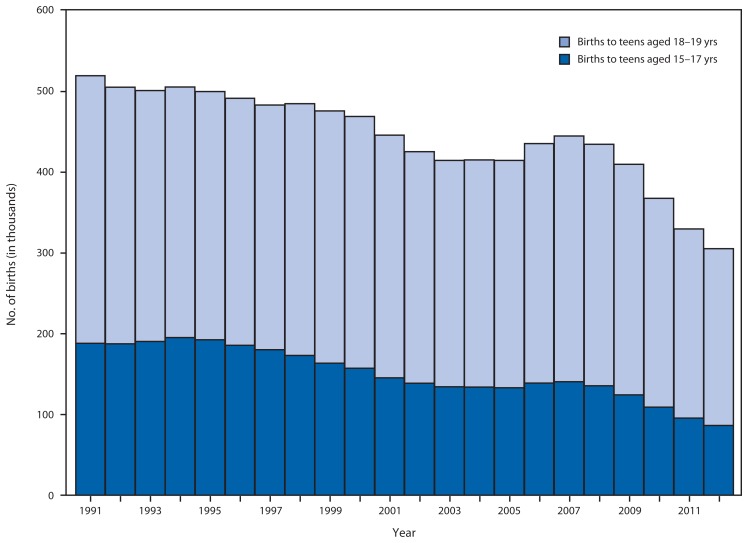
Number of teen births^*^ that were to teens aged 15–17 years compared with teens aged 18–19 years — United States, 1991–2012 **Source:** CDC’s National Center for Health Statistics. National Vital Statistics System data. ^*^ Teen births are defined as births to teens aged 15–19 years.

In 2012, among 305,388 births to teens aged 15–19 years, 86,423 (28.3%) were births to teens aged 15–17 years. The percentage of births to teens aged 15–19 years that were to teens aged 15–17 years declined significantly during the observation period, from 36% in 1991 to 28% in 2012, representing a 22% decrease (p<0.001) (Figure 3).

During 2006–2010, among never-married female teens aged 15–17 years, 27.0% (95% confidence interval [CI] = 23.5%–30.7%) had ever had sex, including 14.6% (CI = 10.3%–20.2%) of those aged 15 years, 28.5% (CI = 22.9%–34.8%) of those aged 16 years, and 38.6% (CI = 33.0%–44.6%) of those aged 17 years. Additionally, 18.0% of teens aged 15–17 years were currently sexually active (CI = 15.2%–21.2%), including 8.0% (CI = 5.2%–12.1%) of those aged 15 years, 16.5% (CI = 13.0%–20.7%) of those aged 16 years, and 29.7% (CI = 24.2%–35.9%) of those aged 17 years. No significant differences by race/ethnicity were observed in the percentage of female teens aged 15–17 years who were sexually experienced or currently sexually active.

Among sexually experienced female teens aged 15–17 years, 22.7% (CI =16.7%–30.2%) used no contraceptive method at first sex; 62.0% (CI = 54.8%–68.7%) used a less effective method, and 15.3% (CI = 11.1%–20.8%) used a moderately or most effective method. Among those teens aged 15–17 years who were at risk for pregnancy at last sex, 40.4% (CI = 32.2%–49.1%) used a moderately or most effective method of birth control, 51.8% (CI = 43.3%–60.2%) used less effective methods, and 7.8% (CI = 4.5%–13.4%) used no method; 1.0% (CI = 0.3%–2.7%) used LARC methods at last sex. Additionally, 57.9% (CI = 49.8%–65.6%) of teens aged 15–17 years at risk for pregnancy received clinical birth control services in the past 12 months: 30.4% (CI = 23.8%–37.8%) received counseling about birth control, 35.2% (CI = 28.4%–42.7%) had a checkup or medical test related to birth control, and 51.7% (CI = 43.8%–59.4%) received a method or a prescription for birth control.

**FIGURE 4:**
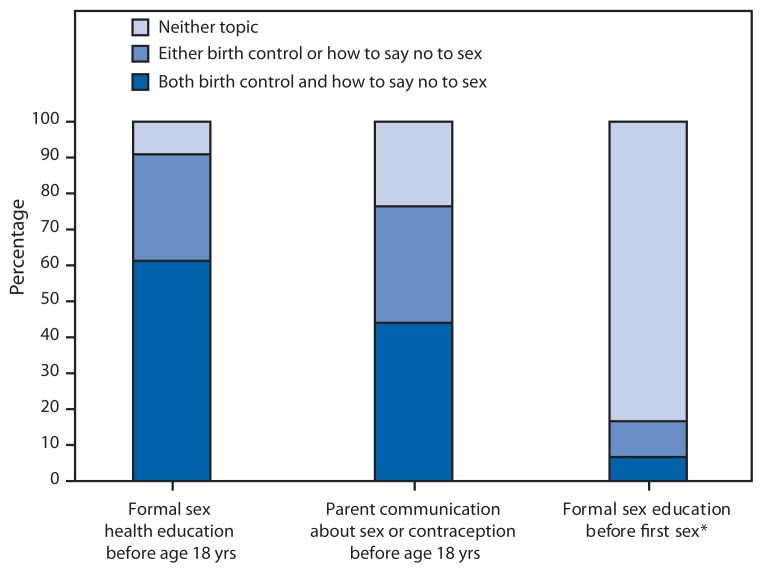
Percentage of never-married females aged 15–17 years who reported receipt of formal sex education and spoke with their parents about sex, by topic discussed — United States, 2006–2010 ^*^ Among teens who reported ever having vaginal sex.

The vast majority of females aged 15–17 years received formal sex education on either birth control or how to say no to sex: 90.9% (CI = 88.1%–93.1%); fewer (61.3% [CI = 56.9%–65.4%]) received information on both topics (Figure 4). In contrast, only 44.0% (CI = 40.0%–48.2%) of female teens aged 15–17 years spoke with their parents about both topics, and 23.6% (CI = 20.7%–26.7%) did not speak with their parents about either topic. Of note, among sexually experienced female teens, 83.3% (CI = 77.2%–88.1%) did not receive formal sex education on these topics before first sex.

## Conclusions and Comment

This report documents sharp declines in the birth rates for teens aged 15–17 years during 1991–2012; in parallel, the proportion of all births to teens aged 15–19 years that were to teens aged 15–17 years also has declined. However, approximately one in four births to a teen aged 15–19 years in 2012 was to a teen aged 15–17 years. There are substantial racial/ethnic disparities in births to younger teens with Hispanics, non-Hispanic blacks, and American Indians/Alaska Natives experiencing disproportionately higher rates (1). Prior research suggests that the overall risk for pregnancy declined 55% for teens aged 15–17 years during 1995–2002, with 23% of the change attributable to a decrease in sexual activity and 77% attributable to changes in contraceptive method use (10). Efforts to prevent pregnancy in this population should take a multifaceted approach, including the promotion of delayed sexual initiation and the use of more effective contraceptive methods.

Numerous evidence-based sexual health education programs have proven effective in delaying sexual initiation or increasing contraceptive use (11). This report suggests that although nearly all younger female teens received some form of sexual health education, only six in 10 reported that information on both birth control and how to say no to sex were covered. Of particular concern is that for most sexually experienced females aged 15–17 years, formal sex education did not precede their first sexual intercourse. This represents a missed opportunity to introduce medically accurate information on abstinence and effective contraceptive use.

Parents are a particularly strong influence on the sexual behavior of teens (12); however, almost a quarter of females aged 15–17 years have not spoken with their parents about how to say no to sex or about methods of birth control. In addition to providing information on normal sexual development, healthy relationships, abstinence, and prevention of pregnancy and sexually transmitted diseases, the quality of parent-child relationships and monitoring teen activities and behaviors are also important in helping a teenager make healthy decisions (13,14).

Although 92% of younger female teens used some form of contraception the last time they had sex, the majority of sexually active female teens aged 15–17 years relied on methods of contraception that are among the least effective during typical use, primarily condoms without simultaneous use of a more effective method. Consistent and correct condom use should be encouraged among sexually active teens to prevent sexually transmitted infections, including human immunodeficiency virus infection. However, during typical use, condoms alone are not highly effective for pregnancy prevention.

Only 1% of sexually active females aged 15–17 years were using LARCs, which have the lowest failure rate among reversible methods of contraception and are safe for use for females of all ages, including teens (15). Previous research from the Contraceptive CHOICE Project, a study that increased use of the most effective birth control methods by providing enhanced contraceptive counseling and removing cost barriers, found that contraceptive failure rates were higher for teens who used the pill, patch, or ring; in contrast, rates of method failure and continuation for non–user dependent methods, such as LARCs, did not differ by age (16). In addition, this intervention demonstrated a reduction in unintended pregnancies and abortions among this age group compared with state and national estimates. However, teens face a number of barriers to LARC use, including cost, lack of health-care provider acceptance for these methods in teens, and lack of awareness of these methods among teens (17).

Use of more effective contraceptive methods by younger teens is limited, in part, because of the need to obtain these contraceptives from a health-care provider; the findings in this report suggest that slightly more than half of sexually active females aged 15–17 years received clinical birth control services in the past 12 months. Efforts that providers can take to increase use of these services by teens include ensuring that clinic services are youth-friendly and providing teen patients with confidential, respectful, and culturally competent services, with convenient office hours, and complete information about the most effective methods of contraception. Laws allowing minors to provide consent for health services and protecting their confidentiality vary by state. Improving confidentiality protections might decrease unintended births for teens (18); professional medical organizations have adopted policy statements that support the provision of confidential care to teens (19). States can consider expansion of eligibility for Medicaid coverage of family planning services to include teens aged <18 years.*

**Key Points**

- Although the birth rate of teens aged 15–17 years declined 63% during the period 1991–2012, about one in four teen births occurred to younger teens (those aged 15–17 years) in 2012.
- About one in four females teens aged 15–17 years have ever had sex. Among those who have, about eight in 10 had not received any formal sex education before the first time they had sex.
- About nine in 10 sexually active younger teens used some form of contraception the last time they had sex. However, only 1% used one of the two most effective reversible methods (i.e., intrauterine devices or implants).
- Strategies to delay sexual initiation and increase the use of the most effective birth control methods include implementing evidence-based youth programs, helping parents talk to their teens about sex and contraception, and making sure that a sexually active younger teen can access reproductive health-care services.

The findings in this report are subject to at least four limitations. First, only data on births were analyzed because more recent data on pregnancies (including miscarriages, stillbirths, and abortions) are not available. Second, births to teens aged <15 years, which also declined during this period, were not analyzed (1). Third, estimates of sexual activity, contraceptive use, and receipt of prevention opportunities are self-reported. Finally, it was not possible to assess the quality and quantity of formal sex education and parent communication about sex.

The findings in this report suggest that fewer young teens are giving birth, the majority of younger female teens are not yet sexually active, and the vast majority of those who are sexually active report use of some method of contraception. Teens need the necessary knowledge, skills, and encouragement to make healthy decisions and engage in healthy behaviors. Continued efforts to prevent teen childbearing among this group should include developmentally appropriate, evidence-based approaches to further delay sexual initiation and introduce the most effective methods of contraception for those who are sexually active.

## Footnotes

* Additional information available at http://www.cdc.gov/stltpublichealth/psr/teenpregnancy/index.html.
