# Errata

**Published:** 2014-04-25

## Vol. 63, No. 14

In the report, “Vital Signs: Births to Teens Aged 15–17 Years — United States, 1991–2012,” multiple errors occurred. In the Introduction, the first sentence should read, “The U.S. teen birth rate has continued to decline, from **61.8** births per 1,000 teens aged 15–19 years in 1991 to an all-time low of 29.4 in 2012 (*1*).” Three errors occurred in the References. Reference 1 should read, “**Martin JA, Hamilton BE, Osterman JK, Curtin SC, Mathews TJ.** Births: final data for 2012. Natl Vital Stat Rep 2013;62(9).” Reference 8 should read, “Lepkowski J, Mosher W, Groves R, West B, Wagner J, Gu H. Responsive design, weighting, and variance estimation in the 2006–2010 National Survey of Family Growth. Vital Health Stat **2013**;2:2–52.” Reference 19 should read, “American Academy of Family Physicians. Adolescents, protecting: ensuring access to care and reporting sexual activity and abuse (position paper). Leawood, KS: American Academy of Family Physicians; **2004**. Available at http://www.aafp.org/about/policies/all/adolescent-protecting.html.”

